# The evaluation of apicectomy without retrograde filling in terms of lesion size localization and approximation to the anatomic structures

**DOI:** 10.4317/medoral.22834

**Published:** 2019-03

**Authors:** Sinan Yasin-Ertem, Hilal Altay, Neda Hasanoglu-Erbasar

**Affiliations:** 1 DDS, PhD. Oral & Maxillofacial Surgery Department Dentistry Faculty. Ankara Yıldırım Beyazıt University; 2 DDS. Oral & Maxillofacial Surgery Department Dentistry Faculty. Ankara Yıldırım Beyazıt University

## Abstract

**Background:**

The purpose of this study was to evaluate of the patients who underwent apical resection. Besides assess the classification of resection side, localization, lesion size, approximation of anatomic structures and the purpose of the apical surgery retrospectively.

**Material and Methods:**

In this stutdy 782 patients and 1191 apical resection applied tooth evaluated. 504 of the patients were famale and 278 were male. Patients age was between 13 and 76 years old and operated between January 2016 and January 2017. The study includes incisor, canine and premolar teeth which had the apical resection as the first time. Operation side evaluated from orthopantomograph and periapical radiographs.

**Results:**

There were 1191 teeth operated and 966 of them in maxilla and 225 of them in mandible. The number of the incisor teeth were 871, 177 were canine, 129 were premolar and one of them was molar. The total amount of 468 patients had operated by just 1 tooth, 454 of the operated teeth had cyst on the operation side. Premolar and molar side 21 of the 93 lesion had approximation with maxillar sinus. On the other hand in maxilla 39 of 569 lesion had approximation with nasal cavity. In mandibula 1 of the 15 lesion, which involved mandibular premolar teeth, had approximation with mental foramen.

**Conclusions:**

Apical resection operation mostly done for one tooth, and the lesion size was less than 10 milimeters. Furthermore apical resection mostly done for incisors cause of odontogenic cyst.

** Key words:**Apical resection, gender, maxillary sinus, mental foramen, odontogenic cyst.

## Introduction

Apical resection is removal of the cyst or infection at the dental root tip together with 1/3 of the bottom part of the dental root. It is a surgical treatment applied usually in case the non-surgical root canal treatment fails. Nevertheless, the evidence for deciding between surgical treatment and non-surgical retreatment is still inadequate. Many studies evaluating the apical resection results have been conducted, and results of such studies are varied. Apical resection is a reasonable surgical intervention failure of which can be ignored in case it presurgical preparation and technical details are planned correctly with correct indication. If apical resection achieves its objective, i.e. it is ensured that the root canal is clogged correctly and apical and periapical pathological foci are removed, no impediment to the bone reformation process remains, thus, the tooth is ensured to stay in the mouth.

Periapical radiographs are the first imaging method to be used in evaluating the apical periodontitis condition after orthograde root canal filling treatment and periapical surgery, in scheduling of the treatment and in follow-up examinations. Post-operative healing condition of apical resection is evaluated by clinical and radiological examination conducted 1 year after the surgery (1).

Surgical approach is usually needed in the presence of infection in the site which cannot be accessed in the apex, in the presence of dentin debris containing bacteria from apical, which causes infection outside the root. The objective of this study is evaluating and qualifying the patients to whom apical resection has been applied in our clinic, and evaluating retrospectively in which cases and on which patients the surgical procedure has been conducted.

## Material and Methods

This is a retrospective study conducted through examining the data of 782 patients indicated with apical resection without retrograde filling, who applied to the Oral and Maxillofacial Surgery clinics of Faculty of Dentistry of Ankara Yıldırım Beyazıt University. The patients who had been operated between January 2016 and January 2017 were evaluated. 1191 apical resections were examined in a total of 782 patients in the study. 504 of these patients were female and 278 were male. Follow-up period of the patient was 1 to 2 years. Incisor, canine and premolar teeth were included in the study, and these were the teeth, which underwent apical resection surgery for the first time. The respective teeth were evaluated by examining the existing panoramic and periapical radiographs.

For panoramic radiographs, A Planmeca ProMax® (Planmeca Oy, Helsinki, Finland) was used for image acquisition with exposure parameters at 66kV and 16mA. Panoramic films were evaluated with the Planmeca Romexis 3.0 software (Planmeca Oy, Helsinki, Finland) program. Periapical radiographs were scanned using a dental X-ray machine (Kodak 2200 Intraoral X-ray System; Carestream Health, Inc., Rochester, NY, USA) operating at 4 mA, 30 cm, and with a total filtration equivalent to 2.5 mm of aluminum, and radiated phosphor plates Digora Optime (Soredex, Tuusula, Finland), and stored. X-ray images were displayed on a 48 cm monitor located in a room receiving low light (190s, Philips, China, resolution 1280 6 1024 pixel, 32 bit).

One observer evaluated and measured the bone defects on the panoramic radiographs individually. Maximum vertical distance was measured from the most coronal to the most apical part of the lesion. Horizontal width of the lesion was measured from the maximum width, which is perpendicular to the vertical measurement line.

45 patients had only panoramic films, 363 patients had only periapical films, and 420 patients had both panoramic and periapical films. The films were examined in a room receiving low light, on the same computer monitor, at the same screen zoom and brightness. The factors leading indication of apical resection were noted as excess canal filling, short canal filling, and the granuloma or cyst formation accompanying to these.

Patients aged 13 to 76 years, to whose at least 1 tooth apical resection operation has been applied, were included in the study. Patients lacking preoperative and/or intraoperative information, with root fracture finding clinically and/or radiographically, undergone periradicular previously, were not included in the study.

Regardless of the number of resected roots, a tooth was considered as a single unit in the evaluation. Evaluation parameters included the information on gender, age, tooth type, lesion size. Lesion size, type of the radiolucent, sufficient, excess or short filling of the root canal filler in the apical of the tooth, may be listed among the radiographical preoperative parameters.

Periapical lesion size was measured in mm with reference to the maximum diameter of the radiolucent area present at the apical or lateral of the root, and categorized as LS<10mmx10mm, 10mmx10mm< LS <10mmX20mm, 10mmX20mm< LS <20mmX20mm, 20mmX20mm< LS <20mmX30mm, 20mmX30mm<LS<30mmx30mm. In cases where the limits of the lesion were unclear, the outermost limit of the pathology was taken as reference.

The final diagnosis of the radicular cysts and granulomas should be made through surgical biopsy and histopathological evaluation. However, since only the radiographs were used when distinguishing the cysts and granulomas, clarity of the bone limit was taken into account while evaluating whether the lesion is a granuloma or cyst. Those with good limits with a radiopaque ring surrounding the radiolucent area around the root of the respective tooth was evaluates as cyst, and in the presence of radiolucent area, they were evaluated as granuloma.

## Results

Panoramic and periapical radiographs were examined for 782 patients (504 female, 278 male) who applied to our clinic and who were indicated with apical resection and operated. A total of 1191 teeth including 966 (81%) superior and 225 (19%) inferior teeth were evaluated. 871 (73.1%) of these teeth were incisor teeth, 177 (15%) were canine teeth, 129 (10.8%) were premolar teeth, and 1 (0.1%) was molar tooth ( Table 1). 1 tooth of 468 patients (59.8%), 2 teeth of 244 patients (31.2%), 3 teeth of 48 patients (6.3%), 4 teeth of 18 patients (2.3%), 5 teeth of 2 patients (0.3%), and 6 teeth of 1 patient (0.1%) were operated ( Table 2). The patients were aged 13 to 76 years, with the mean age 34 years.

**Table 1:**
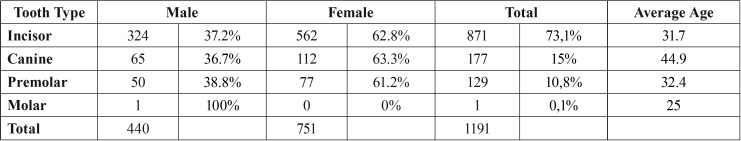
The distribution of tooth type by gender and mean ages.

**Table 2:**
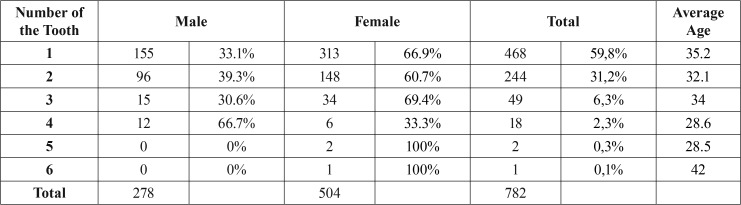
The distribution of the number of the tooth by gender and mean ages.

Cyst formation was observed on 454 of the teeth examined (62.9%). Short canal filling was found on 38 of these teeth, and excess canal filling on 107. Number of the teeth evaluated as granuloma was determined as 267 (36.1%). There is short canal filling on 26 of these teeth and excess canal filling on 61. Since the films of 60 teeth were pertaining to the post-resection, no evaluation could be made about the sufficiency of the canal filling and type of the apical lesion ( Table 3).

**Table 3:**
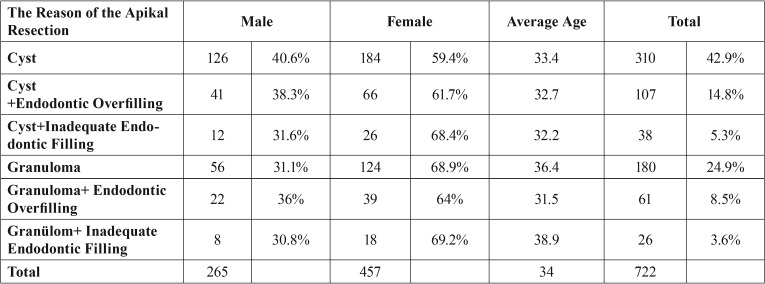
The distribution of the reason of the apical resection by gender and mean ages.

According to the grouping made following the size measurements performed with ruler on the panoramic radiographs under the same screen zoom at the widest vertical and horizontal location point of the lesion, it was found as 402 teeth ≤ 10x10 mm (group1-87.7%); 32 teeth ≥ 10x10 mm / ≤ 10x20 mm (group2-6.9%); 21 teeth ≥ 10x20 mm / ≤ 20x20 mm (group3-4.1%)); 5 teeth ≥ 20x20 mm / ≤ 20x30 mm (group4-1.1%); 1 tooth ≥ 20x30 mm/ ≤ 30x30 mm (gorup5-0.2%) ( Table 4).

**Table 4:**
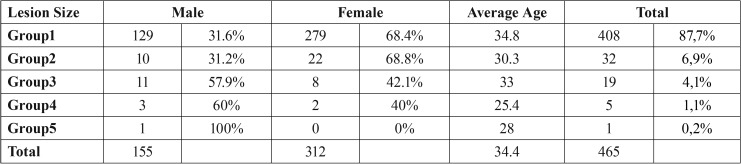
The distribution of the size of the lesion by gender and mean ages.

The teeth were also classified as those with 1/3 apical affected and 2/3 apical affected according to the affected part of the root. According to this classification, 668 lesions affected apical 1/3 of the dental roots and 114 affected 2/3.

Lastly, associations of the superior anterior teeth with nasal cavity, of superior premolar and molar teeth with maxillary sinus, and of inferior premolar teeth with mental foramen, were evaluated. The results were noted as not associated and closely associated. Accordingly, 530 (93.1%) lesions affecting the maxillary incisor teeth were found to be unassociated, and 39 (6.9%) were found to be associated. No association with the maxillary sinus was found in 71 of the lesions in superior premolar and molar site (72.2%), whereas 21 were seen to be associated with maxillary sinus (22.8%). Among 15 lesions affecting the inferior premolar teeth, 14 were located remote to the metal foramen (93.3), whereas 1 was found to be associated with the foramen (6.7%) ( Table 5).

**Table 5:**
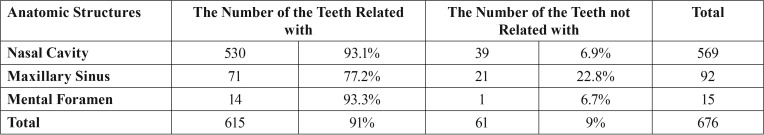
The distribution of the relationship of the tooth with anatomic structures.

## Discussion

In treatment of periapical periodontitis, root canal treatment is always the first step of the treatment. Success rate of this approach was reported as approximately 85% (2). Although endodontic treatment is usually successful, the symptoms may persist or recurrence may be observed in approximately 10% to 15% of the cases. Success rates ranging from 25% to 90% have been reported in the literature for surgical endodontic procedures (3). This difference may be explained with the nature of the study, different sample sizes, controlled periods of the patient, and the criteria used for evaluating the clinical and radiographical parameters of the healing.

Periapical surgery has always been considered as last option before tooth extraction with an unpredictable result. Von Arx *et al.* divided the factors evaluated into three groups as patient-dependent, tooth-dependent and treatment-dependent factors (4). Patient-dependent factors in the literature are usually age and gender. In the statistical study they conducted on 9446 cases, Bumberger *et al.* (5) determined that apical resection operation has been performed in women at the rate of 54% and in men at the rate of 46%, that in terms of mean age, it has been applied most frequently at the ages 21 to 30 years at the rate of 37%. A total of 782 patients were evaluated in our study. According to study results, the rate of women’s undergoing apical resection operation was observed to be 2 folds of men’s. There is a significant difference in favour of women statistically. Since there were 504 female and 278 male patients, rate of female patients in evaluation of apical resection performed to the teeth was higher than rate of male patients, as we expected. However, these rates were found to be directly proportional with number of patients. Namely, it is observed that gender does not lead to any difference between the teeth undergone apical resection.

Besides studies reporting the rate of incidence of periapical cyst at the level of 16-20%, there are also studies where these values have been reported up to 54% (6,7). It can be said that usually cyst-related apical resection operation is performed usually around 30% to 40% in average according to the studies conducted. In the evaluation made by apical resection reasons in our study, it is seen that mean ages are similar in all patient groups and are around 34 years. The most frequent apical resection reason is formation of cyst in more than half (63%) of the patients. One fourth (23%) of the patients were operated due to excess canal filling. Need for operation emerged in 9% of the patients due to short canal filling. In the case where gender difference was examined by the apical resection reason, it was observed not to have any effect.

Radiographic lesion size was suggested as the parameter, which is the most associated with histological diagnosis of the periapical cyst. Previous studies conducted with extracted teeth and apical surgery studies reported a high correlation between size of the periapical lesions and cyst prevalence (8). Moreover, cyst prevalence was reported as 92-100% in the cases with periapical lesion area was higher than 200 mm2 (9). Although there is a tendency of increase of cyst prevalence in big lesions, radiographic size was considered not to be a typical determinant of the periapical lesion (8). In a study conducted by Caliskan *et al.*, when histological diagnosis was considered, 82.2% of the lesions with a diameter of 2–9.9 mm were granulomas and 11.3% were cysts; yet, 51.6 of the lesions with a diameter of 10-20 mm were granulomas and 42% were cysts. It is said that a significant correlation exists between the type and size of the lesion; nevertheless, it is reported that, when considered the limitations of the study such as small sample size, it was concluded that radiographic lesion size is not sufficient for diagnosis of the cyst (10). Barona *et al.* found the success rate as 80% in the lesions sized ≤ 10 mm and 53% in bigger lesions during a follow-up period of 4-10 years (11). It was reported that, when periapical lesion was less than 5 mm, prognosis of periapical surgery was very good (12,13). According to our study; a large rate of the patients like 59.8% have undergone single tooth resection procedure. Around half as much of these patients have undergone operation for 2 teeth. Number of the patients whose 3 or more teeth were operated were observed to be gradually decreasing. According to  Table 4, when the patients were evaluated in terms of lesion size, a great rate of the patients like 87% have lesions less than 1 cm, and number of patients undergoing apical resection decreases as the lesion size increases. As regards distribution by gender, female-male rates were found similar in the same way, only in group 3 and group 4 these rates were found to be significantly higher in favor of men. Moreover, in respect of mean age, mean age of the patients in group 4 was found to be 25 years, slightly lower compared to other groups.

In our study, number of patients decreases as the number of teeth operated due to apical resection increases. Rate of the female patients are similarly high since the number of female patients evaluated was high. This rate changes in favor of men only in patients whose 4 teeth were operated. The reason of this is thought to be related to the lower number of patients.

Bumberger *et al.* (5) stated that this operation is performed mostly on the middle incisor teeth at the maxilla (73.3%) and anterior site (16%). According to the results of our study, a great majority (73%) of the patients to whom apical resection procedure was performed were operated for their incisor teeth, and rates of canine and premolar teeth were too lower and similar. It was also similarly seen in our study that more apical resection procedures were performed to the teeth at the maxilla (81%), and at a lower rate to the mandibula (19%).

Closeness of maxillary sinus is not a contraindication for periapical surgery in superior premolars and molars (14,15). Despite this, Kreisler *et al.* have found a marked drop in the success rate in the teeth with postoperative oroantral fistula after 6-12 months.16 When relationship of the patients with nasal cavity, sinus and mental foramen were evaluated, presence of such relationship was found in 9% of them. When these relationships were evaluated, in terms of anatomic neighbourhoods in patients undergone apical resection, maxillary sinus was found to be related most at the rate of 22%, followed by nasal cavity and mental foramen with similar rates.

According to the results of our study, although age and gender rates show similarity with the literature, cyst rate is seen to be slightly higher compared to the literature. Besides this, the patients undergone apical resection have been operated usually for one tooth and lesion size is seen to be smaller. Moreover, the operation has been often performed to incisor teeth, but not due to presence of cyst.
